# A reliable and quick method for screening alternative splicing variants for low-abundance genes

**DOI:** 10.1371/journal.pone.0305201

**Published:** 2024-06-27

**Authors:** Yanchun Zhang, Wubin Qu, Ruifen Yan, Huqi Liu, Chenggang Zhang, Zhihui Li, Guofu Dong

**Affiliations:** 1 Department of Blood Transfusion Medicine, The Seventh Medical Center of PLA General Hospital, Beijing, China; 2 Laboratory of Electromagnetic Biological Effects, Beijing Institute of Radiation and Medicine, Beijing, China; 3 College of Life Science, Northwest Agriculture and Forest University, Yangling, China; Hainan University, CHINA

## Abstract

Alternative splicing (AS) is a universal phenomenon in eukaryotes, and it is still challenging to identify AS events. Several methods have been developed to identify AS events, such as expressed sequence tags (EST), microarrays and RNA-seq. However, EST has limitations in identifying low-abundance genes, while microarray and RNA-seq are high-throughput technologies, and PCR-based technology is needed for validation. To overcome the limitations of EST and shortcomings of high-throughput technologies, we established a method to identify AS events, especially for low-abundance genes, by reverse transcription (RT) PCR with gene-specific primers (GSPs) followed by nested PCR. This process includes two major steps: 1) the use of GSPs to amplify as long as the specific gene segment and 2) multiple rounds of nested PCR to screen the AS and confirm the unknown splicing variants. With this method, we successfully identified three new splicing variants, namely, GenBank Accession No. HM623886 for the *bdnf* gene (GenBank GeneID: 12064), GenBank Accession No. JF417977 for the *trkc* gene (GenBank GeneID: 18213) and GenBank Accession No. HM623888 for the *glb-18* gene (GenBank GeneID: 172485). In addition to its reliability and simplicity, the method is also cost-effective and labor-intensive. In conclusion, we developed an RT-nested PCR method using gene-specific primers to efficiently identify known and novel AS variants. This approach overcomes the limitations of existing methods for detecting rare transcripts. By enabling the discovery of new isoforms, especially for low-abundance genes, this technique can aid research into aberrant splicing in disease. Future studies can apply this method to uncover AS variants involved in cancer, neurodegeneration, and other splicing-related disorders.

## 1. Introduction

Alternative splicing (AS) is a universal phenomenon in eukaryotes. Some studies have revealed that more than 80% [1] of eukaryotic genes are alternatively spliced, and even 95% [2] of human genes are alternatively spliced. AS, which can increase the diversity and complexity of genes and proteins, is closely associated with the development of cancer [3–5] and nervous system diseases [6–8]. Therefore, establishing a valuable and highly efficient approach for identifying splice variants is important for studying splicing-related diseases. A previous study demonstrated several techniques for novel splice variant identification, including bioinformatics and molecular methods, such as expressed sequence tag (EST) [9, 10], microarray [11–13], RNA-seq [14, 15], 5’ RACE and genomic mapping [16]. However, there are several disadvantages in EST data, such as 3’ deflection, a 2%-5% error rate, cDNA contamination from vector sequences and extracellular mRNA sources, genomic contamination, and low-abundance gene insensitivity. Microarray technologies also have their inevitable drawbacks, including limited array coverage and nonspecific array hybridization. For example, microarray analysis is the standard method for analyzing miRNA expression profiles; however, it has several disadvantages, including its limited detection of miRNAs. Furthermore, microarray methods lack the dynamic range to detect and quantify low-abundance transcripts, but deep sequencing can identify miRNAs that are expressed at levels that fall below the detectable threshold of the microarray. In addition, deep sequencing eliminates background problems that result from cross-hybridization in microarrays, thus facilitating interpretation of the signal and eliminating the nonlinear data manipulation steps required by microarrays [17]. At present, RNA-seq has generally been used in AS studies, but this technique is very expensive. For example, due to the overall costs of RNA-seq, technical replicates are usually omitted, especially in observational studies that already include a large number of biological replicates. For example, high reproducibility of RNA-seq analyses has been reported, and the use of technical replicates at the same study points could improve the statistical power of an experiment [18]. To date, RNA-Seq data obtained with short-read sequencers have been selected for transcriptome analysis due to their high fidelity, high coverage, and single-nucleotide resolution. However, it is difficult to accurately characterize full-length transcripts using short-read sequencers due to limitations in read length [19].

In summary, these approaches are enormously limited due to their low reliability, poor positive rate, need for experimental validation, and high cost and tedious operation. To address these issues, we established a method for screening AS variants, especially for low-abundance genes of a known gene, using gene-specific primers (GSPs) for RT followed by nested PCR (nPCR), which can overcome these difficulties and provides an advantageous combination of specificity, efficiency, accuracy and usability. Compared with other techniques for detecting new variants, this research method specifically screens for new variants of a known gene rather than extensively screening for new variant genes in large quantities. It is more targeted and enriched. This method is also important for functional studies of eukaryotic genes, especially low-abundance genes, and for understanding the mechanism of aberrant splicing-related diseases.

## 2. Description of the method

### 2.1 Obtaining the longest transcribed RNAs from GSPs located at the 3’-end of the gene

To amplify as long as the specific gene segment, GSPs were used as RT primers rather than universal primers (random primer and oligo(dT)_20_) and were designed at the distal end of the gene sequence, which is located in the corresponding exons. The advantage of GSPs is the enrichment of target gene RNA transcripts, which provides the following nPCR products with templates.

### 2.2. Determination of the reducing pattern of the size of amplicons by multiple rounds of nested PCR

Multiple upstream and downstream GSPs of every splice variant of target genes were designed according to known gene structure. The upstream and downstream primers (2–3 primers each terminal) were designed from the outer to the inner regions of the 5’- and 3’-extremmitates, and it is better to cross introns in the case of genomic contamination. The *bdnf* splice variant 1 in *BALB/c* mouse brain tissue was used as an example to describe nPCR. There are five rounds of PCR for identifying splice variant 1 of *bdnf*. Most of the lateral GSPs (tv1-5’GSP1 and 3’GSP1) were used to amplify specified splicing variants with cDNA as a template in the first round of nPCR. Then, the 5’-GSPs were locked, while the 3’-GSPs were gradually chosen from the outer to the inner regions. Finally, by immobilizing 3’-GSPs, 5’-GSPs were selected step by step for amplification. Every round of PCR was performed using the same program but with a 1:10 dilution of the former round products as the template, rather than the template of the first round cDNA. Eventually, each round of PCR amplicons was separated on 1.5% agarose gels supplemented with ethidium bromide. At this time, specific and nonspecific bands were observed under UV light, and we can determine preliminarily whether new variants existed.

### 2.3. Validation of the new variants by sequencing

Decreasing patterns of electrophoresis strips were identified for known and unknown variants by T-A cloning, sequencing, bioinformatics analysis, and further determination of the AS forms of the new variants.

## 3. Validation of the methods

### 3.1. Culture and synchronization of *C*. *elegans*

The wild-type N2 strains of *C*. *elegans* (Caenorhabditis Genetics Center (CGC), USA) were cultured in 90 mm petri dishes with nematode growth medium (NGM). Adult nematodes were collected and treated with 5 ml of nematode lysate containing 400 μl of 8% sodium hypochlorite, 80 μl of 10 M NaOH and 4.52 ml of M9 buffer. The solution was shaken vigorously by hand for approximately 10 min. At the same time, the state of the lysate was observed. The lysate solution was centrifuged for 1 min at 1200 rpm to collect the eggs, which were subsequently washed three times with M9 buffer. The eggs were inoculated on NGM agar plates without *E*. *coli* OP50 for 16 h at 20°C (± 0.1°C) to hatch L1 larvae. Synchronized L1 larvae were then collected and placed on 90 mm NGM agar plates with *E*. *coli* OP50 seeded [20].

### 3.2. Preparation of total RNA from different species

The *BALB/c* and *C57BL/6J* mice (18–20 g) were obtained from Beijing Weitong Lihua Experimental Animal Technology Co., Ltd. [SCXK (Beijing)-2015-0001]. During the study, mice were kept in the laboratory environment under 12/12 h day/night periods, 22°C room temperature, and 60% relative humidity. The disposal of animals during the experiment gives humanitarian care according to the 3R principle used by experimental animals. After anesthesia was intraperitoneally injected with 1% pentobarbital sodium (50 mg/kg), the animals were euthanized by neck amputation, and their brain tissues were prepared according to previous methods [21]. When the worms grew to the L4 stage after synchronization, they were washed and collected from the plates with M9 buffer and subsequently cleaned three times [22]. Total RNA was extracted using a total RNA kit (OMEGA, Japan).

### 3.3. Design of GSPs

According to the principle of our method, to aim directly at *bdnf* of *BALB/c* mice, *trkc* of *C57BL/6J* mice and *glb-18* of *C*. *elegans*, multiple pairs of GSPs for RT and nPCR were designed by MPprimer [23] and evaluated by MFEprimer [24], as shown in Table 1 and Table 2.

**Table 1:** The GSPs used in this study. RT-primer (GSPs) primers.

		
*bdnf in BALB/c mouse*	GSP	5’-AGCAACAGGCCTGCTGCCAT-3’
*trkc* in *C57BL/6J* mouse	GSP	5’-AGCACGGGCCACAGCTTAAGT-3’
*glb-18 in C*. *elegans*	GSP	5’-GGCGGAAAACGATTGACACCTTTCA-3’

**Table 2:** nPCR (GSPs) primers.

Gene		Primers(5’-3’)
*bdnf in BALB/c mouse*	tv1-5’GSP1	5’-CAGTAGCCGGCTGGTGCAGA-3’
	tv1-5’GSP2	5’-TGCTTCAGGAAACGCCCGCT-3’
	tv1-5’GSP3	5’-ACGTGTCTCTCAGAATGAGGGCGT-3’
	tv2-5’GSP1	5’-TGGCAAAGCCATCCACACGTG-3’
	tv2-5’GSP2	5’-CGAGGTTCGGCTCACACCGA-3’
	tv2-5’GSP3	5’-AGCCCCAGTTTGGTCCCCTC-3’
	tv3-5’GSP1	5’-AGAGGACTGCTCTCGCTGCC-3’
	tv3-5’GSP2	5’-GCTTCTCGCTGAAGGCGTGC-3’
	tv3-5’GSP3	5’-CCACCAAAGACTCGCCCCCT-3’
	3’GSP1	5’-ATGCCCCTGCAGCCTTCCTT-3’
	3’GSP2	5’-TGGGCCGAACCTTCTGGTCC-3’
	3’GSP3	5’-GAGTCCCATGGGTCCGCACA-3’
*trkc* in *C57BL/6J* mouse	5’-GSP1	5’-CCCTCTCCTGGAAGGGCAGG-3’
	5’-GSP2	5’-AGAGAACTGGCGAGGCCTGC-3’
	3’-GSP1	5’-ACACGGCCTTGGGTGATGCA-3’
	3’-GSP2	5’-CATCCAGCGGATGGGGAGCA-3’
*glb-18 in C*. *elegans*	5’GSP1	5’-TGCCGTCTGCTGCTCGTCAA-3’
	3’GSP1	5’-GGCGGAAAACGATTGACACCTTTCA-3’
	3’GSP2	5’-TCCTCCACACCGTCACTGCG-3’
	3’GSP3	5’-TCGGTCATAATGGAGAGACGGTGTT-3’

The main steps for designing GSPs are outlined below, using the design of three primer pairs for a target transcript of 2000 bp as an example. This approach meticulously controls the "INCLUDED REGION" and "PRIMER PRODUCT SIZE RANGE" parameters in MPprimer, ensuring precision from the outermost to the innermost primers.

The following principles were used to design the RT‒PCR primers:

RT primer (GSP) design: GSP extends from the 3 ’RACE end to 50~60 bp for known genes.

Design principles of the nPCR primers:

Outer GSP1 Design: Convert "INCLUDED_REGION = 1,100 900,100" to select the forward primer of GSP1 from the start to the 100 bp position and the reverse primer from 900 bp to the end. Additionally, we set "PRIMER_PRODUCT SIZE_RANGE = 800–1000" to maintain the desired product size within a specific range.

Middle GSP2 Design: Specify "INCLUDED_REGION = 100,100 800,100" and "PRIMER PRODUCT SIZE RANGE = 600–800" for the design of GSP2, strategically positioning it within the transcript.

Inner GSP3 Design: For GSP3, set "INCLUDED REGION = 200, 100 ’700, 100" with "PRIMER PRODUCT SIZE RANGE = 400–600", focusing on a more central region of the transcript.

Crucially, adjusting the "INCLUDED REGlON" to include exon‒intron junction sites ensures that primers spanning these regions do not amplify genomic DNA. Selecting different exons for GSP2 or GSP3 offers an effective strategy for discovering new variants. Finally, it is essential to confirm all primers with the MFE primer to ensure that they produce the expected amplicons without any off-target amplification.

### 3.4. GSP-dependent reverse transcription

The cDNA was prepared by RT using a ReverTra Ace-α- (Code No. FSK-100) kit (TOYOBO, Japan) in a Professional Thermocycler (Biometra, Germany) as follows: 0.5 nM self-designed RT-primer (GSPs) (Table 1), 1 μg of total RNA, and up to 12 μl of RNase-free H_2_O in a PCR tube for 5 min at 65°C. After the above procedure, the mixture was placed on ice, and 5× RT Buffer (MgCl_2_ plus), 1 mM dNTP mixture, 10 units of RNase inhibitor and 1 μl of ReverTra Ace were immediately added. The running program was 42°C for 40 min, 85°C for 5 min, and 4°C for 5 min. The cDNA was stored at 4°C until use.

### 3.5. nPCR

**Fig 1:**
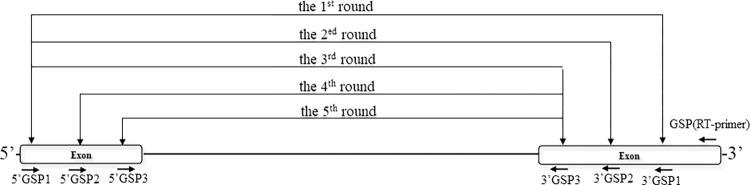
Schematic diagram of nPCR. In the figure, all GSPs are used in *bdnf* splice variant 1. RT-primer was used only for reverse transcription. There were five rounds of PCR, which produced 1258 bp, 900 bp, 783 bp, 523 bp and 430 bp. The primer combinations used were 5’GSP1+3’GSP1, 5’GSP1+3’GSP2, 5’GSP1+3’GSP3, 5’GSP2+3’GSP3, and 5’GSP3+3’GSP3.

nPCR was performed to screen for AS events after the RT procedure. There were five rounds of nPCR for *bdnf* gene detection (Fig 1). The combinations of variant 1 primers were tv1-5’GSP1+3’GSP1, tv1-5’GSP1+3’GSP2, tv1-5’GSP1+3’GSP3, tv1-5’GSP2+3’GSP3, and tv1-5’GSP3+3’GSP3 (Table 2). The variant 2 primers used were tv2-5’GSP1+3’GSP1, tv2-5’GSP1+3’GSP2, tv2-5’GSP1+3’GSP3, tv2-5’GSP2+3’GSP3, and tv2-5’GSP3+3’GSP3. The variant 3 primers used were tv3-5’GSP1+3’GSP1, tv3-5’GSP1+3’GSP2, tv3-5’GSP1+3’GSP3, tv3-5’GSP2+3’GSP3, and tv3-5’GSP3+3’GSP3. There were three rounds of nPCR for *trkc* detection, and the primers used were 5’GSP1+ 3’GSP1, 5’GSP1+3’GSP2, and 5’GSP2+3’GSP2. There were three rounds of nPCR for *glb-18* detection, and the primers used for assembly were 5’GSP1+3’GSP1, 5’GSP1+3’GSP2, and 5’GSP1+3’GSP3.

For example, in *bdnf* gene detection, following PCR amplification using GSPs, 0.5 μl of cDNA was used for the first-round amplification of 35 cycles of melting temperature (45 sec at 95°C), annealing temperature (45 sec at 62°C) and extension time (1 min at 72°C) in a professional thermocycler (Biometra, Germany).

The 12.5 μl reaction mixture contained 10×Ex Taq Buffer (Mg^2+^ plus), 200 μM of each dNTP, 0.2 nM of forward primer tv1-5’GSP1, 0.2 nM of reverse primer 3’GSP1, 0.315 units of TaKaRa Ex Taq, 0.5 μl of cDNA and 9.187 μl of double distilled water (ddH_2_O). The second-round reaction was performed using the same program but with a 1:10 dilution of the first-round reaction as the template, and the primers used were tv1-5’GSP1 and 3’GSP2. Similarly, the third, fourth, and fifth rounds of PCR were performed using the same program but with a 1:10 dilution of the second, third, and fourth rounds of reaction as the template, and the corresponding primers were used.

The inspection of other variants of *trkc* and *glb-18* was performed using the same program, but the primers were adjusted. The PCR products were separated on 1.5% agarose gels supplemented with ethidium bromide. DNA was visualized under UV light. All of the experiments were repeated at least three times.

### 3.6. Verification of variants by sequencing

For the purpose of verification of splice variants, the sequencing results were analyzed after removing the vector sequence using the BLAST program (http://blast.ncbi.nlm.nih.gov/Blast.cgi). The gene structures of *bdnf* in *BALB/c* mice, *trckc* in *C57BL/6J* mice and *glb-18* in *C*. *elegans* were subsequently verified via BLAST results and GenBank information in the NCBI RefSeq database. The new variant experiment was repeated three times for verification.

## 4. Results

### 4.1 Identification of known splice variants of the *bdnf* gene in *BALB/c* mice

**Fig 2:**
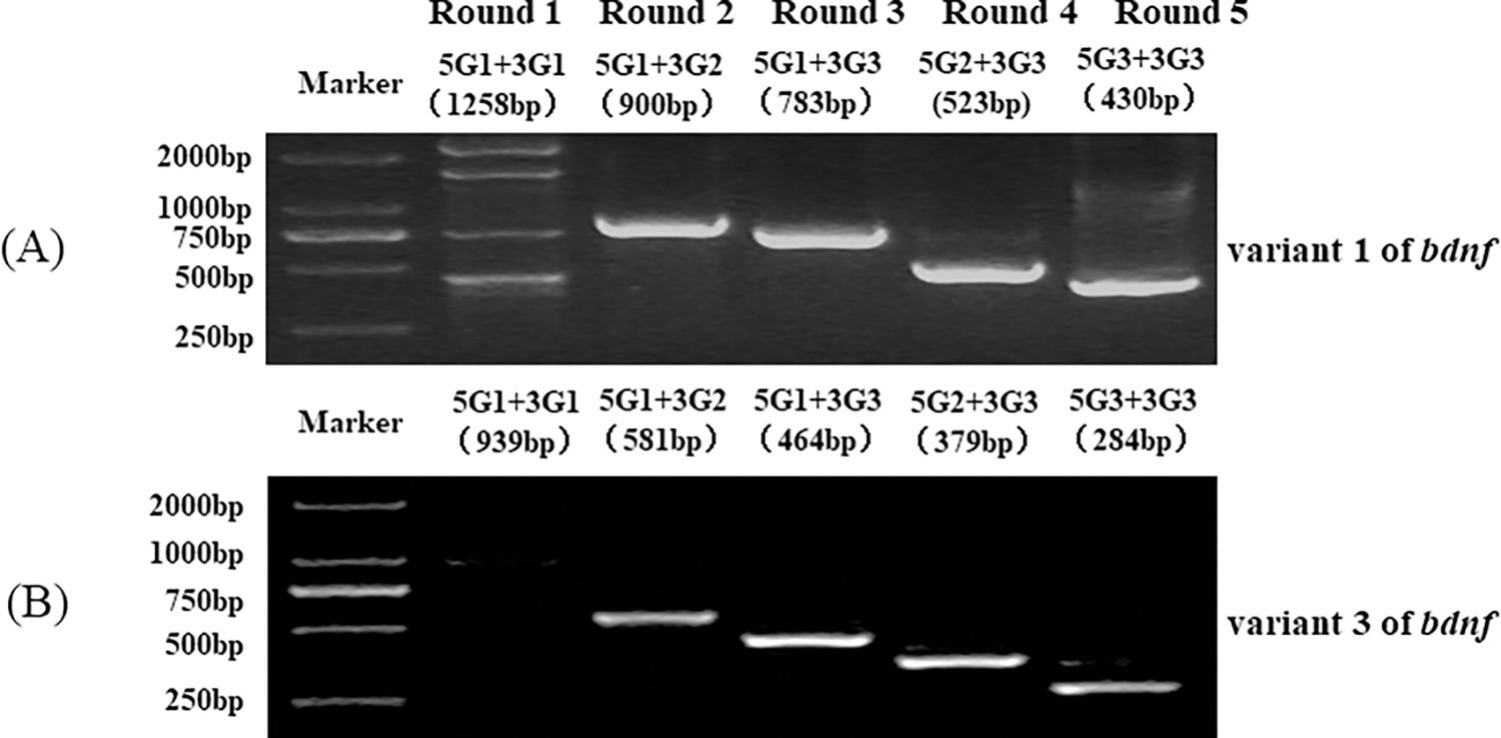
nPCR results of *bdnf* splice variant 1 and variant 3 in *BALB/c* mice. (A) nPCR results of *bdnf* splice variant 1. (B) nPCR results of *bdnf* splice variant 3.

To validate the feasibility of splice variants by using RT and nPCR with GSPs, known splicing variant 1 (Fig 2A) and variant 3 (Fig 2B) of the *bdnf* gene in *BALB/c* mice were identified. The results showed that this method can identify these two variants (Fig 2). As expected, there was one specific electrophoresis band with a decreasing pattern of products.

### 4.2 Screening of a new splice variant of the *bdnf* gene in *BALB/c* mice

**Fig 3:**
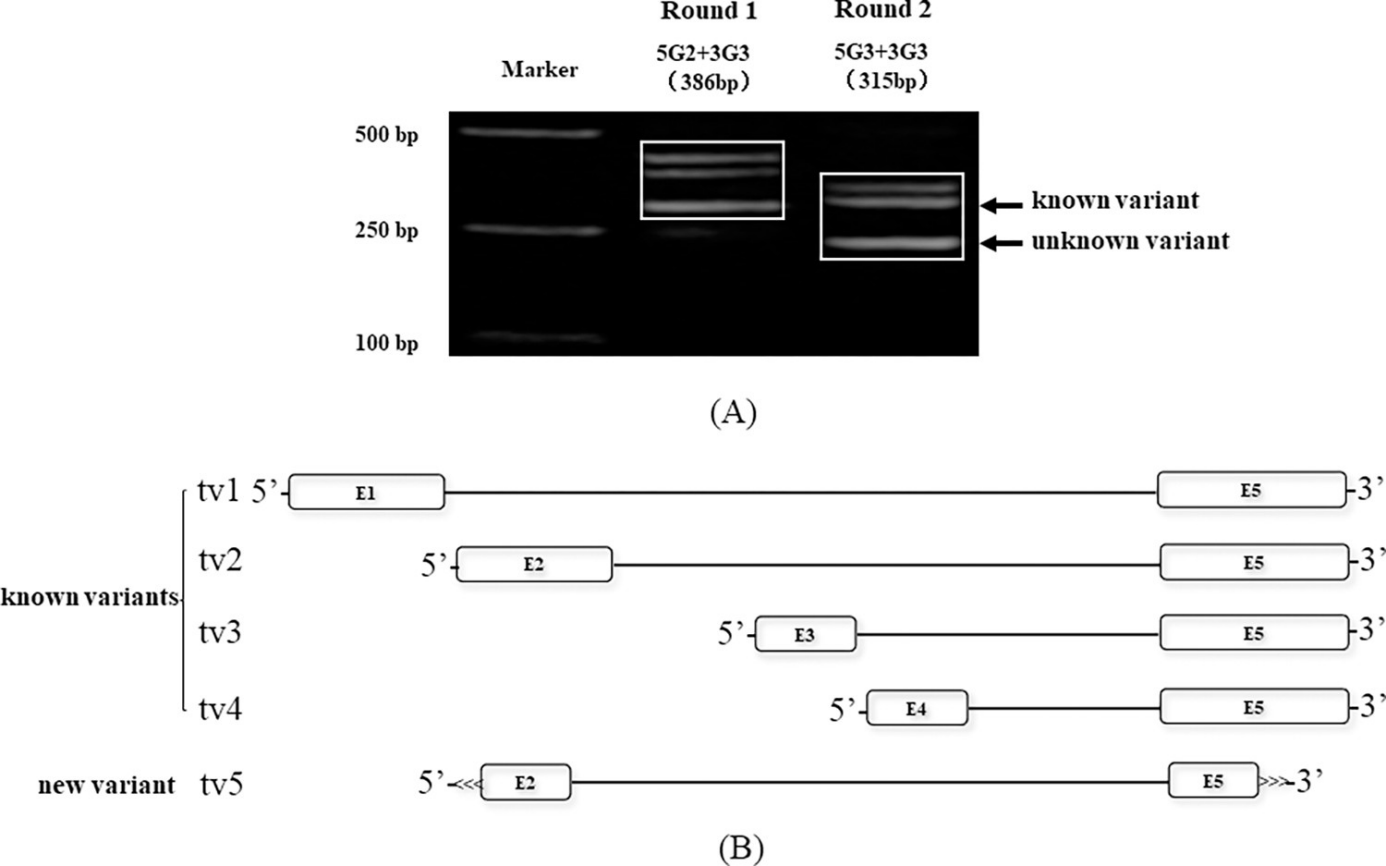
(A) nPCR results of *bdnf* variant 2 in *BALB/c* mice. Sequencing and analysis revealed that the second variant was known, while the third variant was new. (B) Gene structure of *bdnf* in *BALB/c* mice.

The nPCR results of variant 2 displayed three DNA bands with a diminishing pattern (Fig 3A). Direct sequencing results and BLAST analysis revealed that the second band in which the arrow was directed was the known variant, while the third band was the unknown variant, which has high homology with the *bdnf* gene, particularly variant 2 (Fig 3A). We mapped the gene structure of every *bdnf* variant according to its GenBank information in the NCBI database, as shown in Fig 3B. In addition, the unknown variant was deposited in the GenBank database under accession no. HM623886.

### 4.3 Screening of a new splice variant of the *trkc* gene in *C57BL/6J* mice

**Fig 4:**
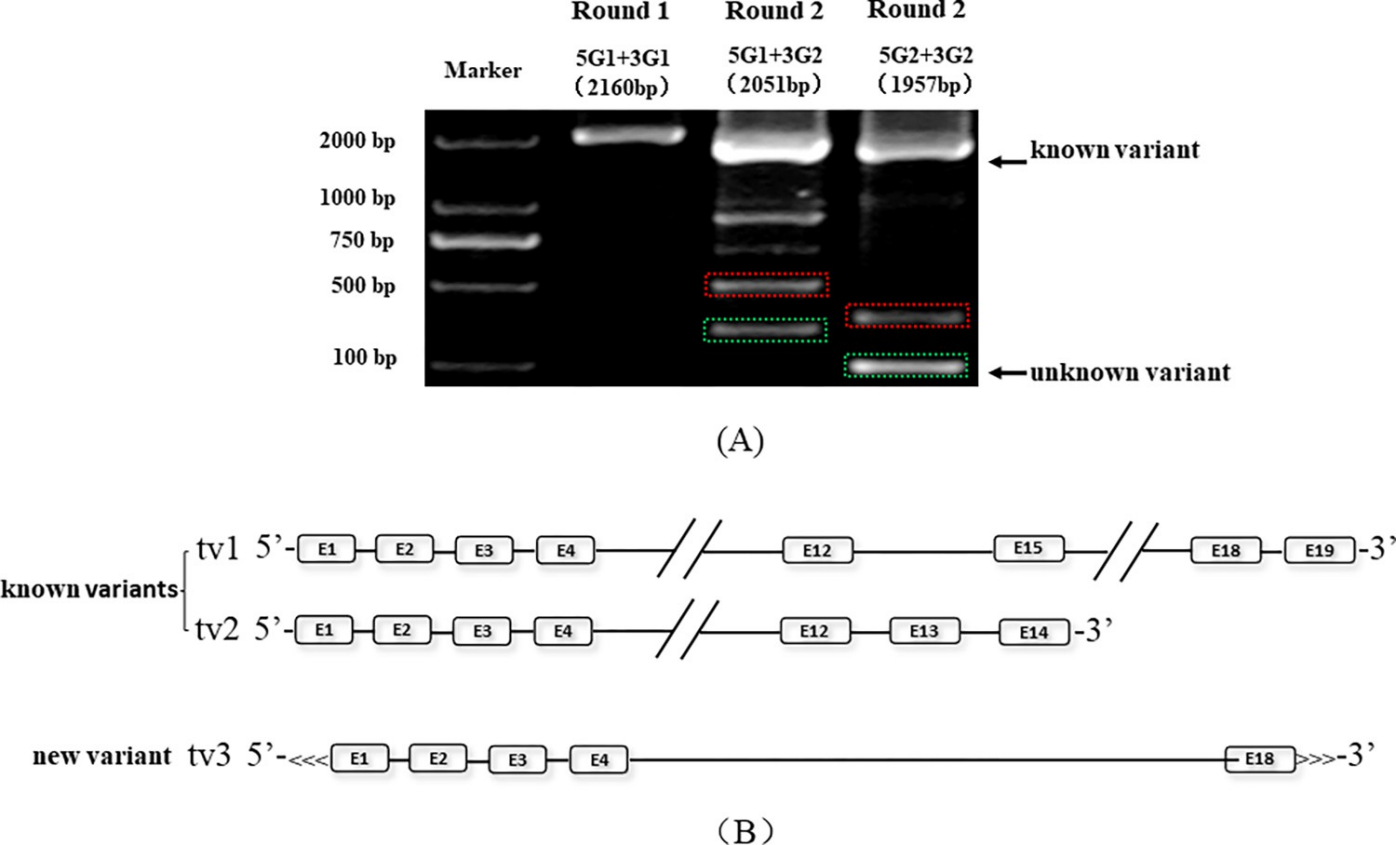
(A) Results of nPCR analysis of *trkc* in *C57BL/6J* mice. In addition to the expected bands, two bands showed a decreasing pattern. Sequencing and analysis revealed that the bands in the dotted box region represented a new variant. (B) Gene structure of the *trkc* gene in *C57BL/6J* mice.

The identification results of the *trkc* gene are shown in Fig 4A. Three target bands with decreasing sizes of 2160 bp, 2051 bp and 1957 bp can be obtained by nPCR (as shown by the arrows in Fig 4A, Lane 2, Lane 3 and Lane 4, respectively). Known variant 1 of the *trkc* gene can also be identified by using RT‒PCR and nPCR with GSPs.

For the nucleic acid fragments shorter than 500 bp in Fig 4 (marked by a solid frame and dotted box in Lane 3), the length of the band in the dotted box was 335 bp. Compared with that in the mouse RefSeq database, the nucleic acid fragment sequence in the RefSeq database was highly similar to that in the *trkc* gene (99%), especially with variant 1. Through in-depth analysis, it was found that the 1–298 bp sequence was consistent with the 222–519 bp sequence in *trkc* gene splice variant 1 and that the 299–334 bp sequence was consistent with the 2189–2224 bp sequence in *trkc* gene splice variant 1, indicating that the 1669 bp sequence missing between the 520–2188 bp sequence in *trkc* gene splice variant 1. Therefore, it is a new splice variant of the *trkc* gene and was determined to be variant 3. The nucleic acid fragment in the solid frame of Fig 4 was identified as a nonspecific amplification band by sequencing.

We mapped the gene structure of each *trkc* variant according to its GenBank information in the NCBI database, as shown in Fig 4B. The cDNA sequence of this variant has been submitted to the GenBank database under sequence acceptance number JF417977.

### 4.4 Screening of a new splice variant of the *glb-8* gene in *C*. *elegans*

**Fig 5:**
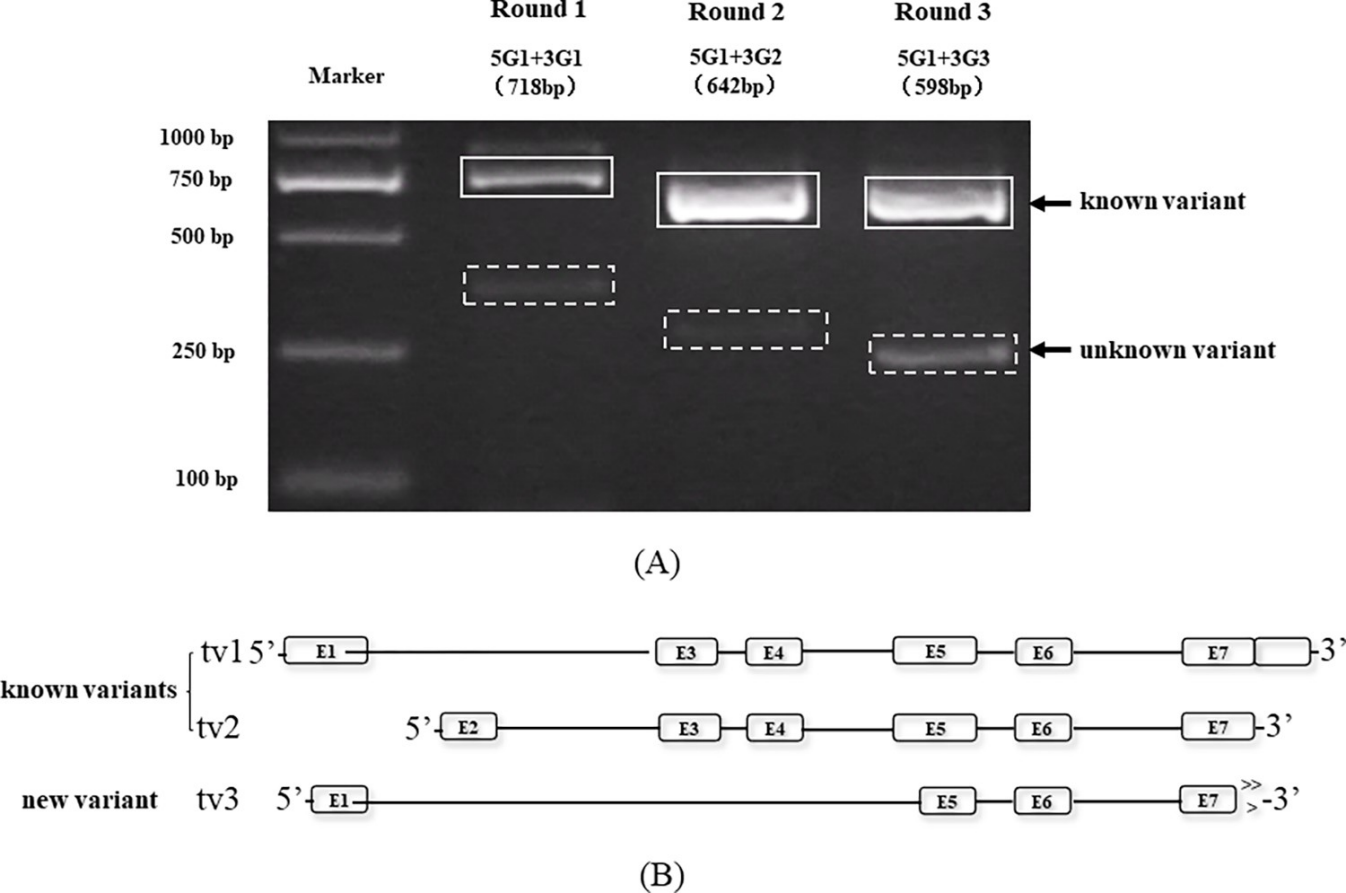
(A) Results of nPCR analysis of *glb-18* in *C*. *elegans*. In addition to the expected bands, there is a decreasing band pattern. Sequencing and analysis revealed that it is a new variant. (B) Gene structure of the *glb-18* gene in *C*. *elegans*.

We further asked whether our approach worked well for other species. Likewise, in *C*. *elegans*, *glb-18* also had strips with a diminishing pattern, and other than the expected strips, there were unexpected strips with the same diminishing pattern, which suggested that it was probably a new variant (Fig 5A). Sequencing results and BLAST analysis revealed that the band in the solid line was the known variant, while the band in the dashed line had high homology with *glb-18*, particularly variant 1. Similarly, we mapped the gene structure of each *glb-18* variant according to its GenBank information in the NCBI database, as shown in Fig 5B. The unknown variant is missing exon 2, exon 3, exon 4, and part of exon 1 and exon 5 and was deposited in GenBank (accession no. HM623888).

## 5. Discussion

AS, a popular research topic, is common in organisms and is closely associated with various diseases. Currently, existing methods for identifying splice variants, such as EST [9, 10], microarray [11–13], RNA-seq [14, 15], 5’RACE and genomic mapping [16], have defects. 5’RACE requires the design of the gene-specific primer GSP-RT for reverse transcription. The first-strand (-) cDNA was obtained via reverse transcription of GSP-RT mRNA, and then the poly(A) tail was added to the cDNA 5’ end using deoxyribonucleic acid terminal transferase and dATP. The second-strand (+) cDNA was synthesized using anchored primers. However, 5′RACE efforts are characterized by several drawbacks, such as the requirement of excess initial RNA material as well as the need for multiple enzymatic procedures, making the entire process extremely time-consuming [25]. High-resolution melting analysis is another option for detecting small variants. This requires improved instrumentation and the application of new DNA-binding dyes. Simple intercalating dyes do not saturate the entire dsDNA fragment. This leads to dye redistribution during melting, which decreases the specificity of discrimination [26]. The dynamic range of RNA-Seq data is wider, and the number of false-positives may be smaller, which means that the data repeatability of RNA-Seq data should be greater than that of chip data. RNA-Seq can detect all RNA in a sample, which is an advantage for identifying novel transcripts in cells, but at the same time, its disadvantage is that it detects total RNA, and a large portion of RNA in cells comes from ribosomes and mitochondria. This limits the number of reads for other RNAs and the accuracy of their expression levels [27]. Existing methods for identifying AS variants have several limitations. These include low reliability, poor positive rates, lack of experimental validation, genomic DNA contamination, inability to detect low-abundance transcripts, high cost, and tedious protocols. Furthermore, most methods rely on universal RT primers such as random hexamers and oligo(dT)_20_, followed by routine PCR for verification. This process fails to enrich for rare transcripts. Compared with other techniques for detecting new variants, this research method specifically screens for new variants of a known gene rather than extensively screening for new variant genes in large quantities. It is more targeted and enriched. Because random primer and oligo(dT)_20_ are used for RT experiments, they can be combined with all mRNA or noncoding RNA in the RNA template and reverse transcribed into cDNA [28]. It is difficult to capture low-abundance mRNAs, and alternative exons are rarely found via routine PCR. Nevertheless, in this study, we established a method for screening gene splice variants using GSPs for RT and nPCR techniques that can redeem this fault and provide experimental testing for bioinformatics approaches. The key aim of this study was to use primer design software (MPprimer and MFEprimer) developed by our laboratory to design GSPs instead of ordinary random primers and oligo(dT)_20_. In addition, multiple rounds of nPCR using GSPs can quickly screen for splice variants and identify unknown variants, and electrophoresis strips with diminishing patterns also greatly increase amplification product specificity. For example, through the above methods, we screened three new variants, namely, *bdnf* and *trkc* gene variants from mice and *glb-18* variants from *C*. *elegans*, all of which are low-abundance genes. The splice variants are important for mechanistic research on aberrant splicing-related diseases and can also aid in the diagnosis and treatment of these diseases with reliable technological evidence. Therefore, the method of screening splicing variants in this study has great application prospects. However, this method has inevitable disadvantages; for example, only a fraction of subsequences can be obtained rather than their total length, and this is not possible for high-flux sequences. At present, our laboratory is being engaged in mPCR, including primer design [23] and evaluation [24], as well as specific experimental procedures. In the future, multiplex PCR (mPCR) will be applied to this method, and multiple variants of one gene or several genes could be identified simultaneously.

Taken together, the current method is a quick, reliable, low-abundance gene sensitive, cost-effective and labor intensive approach for screening alternative splicing variants of known genes that can cover known and unknown splice variants. This approach overcomes the limitations of existing methods for detecting rare transcripts. By enabling the discovery of new isoforms, especially for low-abundance genes, this technique can aid research into aberrant splicing in disease. Future studies can apply this method to uncover AS variants involved in cancer, neurodegeneration, and other splicing-related disorders.

## Supporting information

S1 Fig (JPG)

S2 Fig (JPG)

S3 Fig (JPG)

S4 Fig (JPG)

S5 Fig (JPG)
