# Supplementary figures and images for: A reliable and quick method for screening alternative splicing variants for low-abundance genes
Source: PLoS One. 2024 Jun 27;19(6):e0305201. doi: 10.1371/journal.pone.0305201 (PMC11210779; doi:10.1371/journal.pone.0305201)

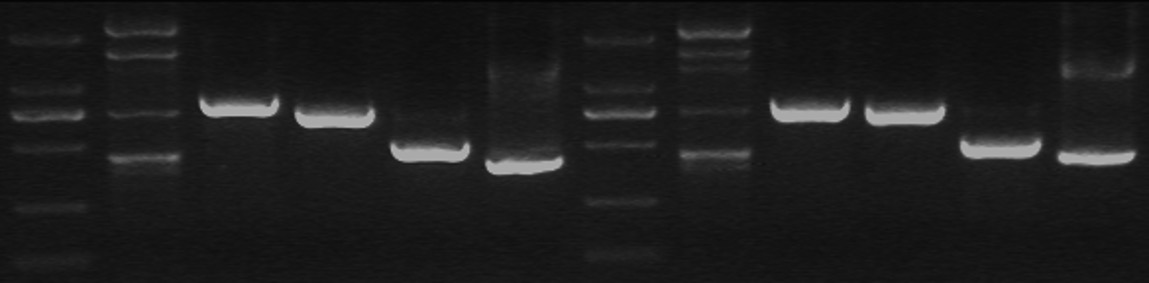

Supplement: S1 Fig — (JPG) [file pone.0305201.s001.jpg]

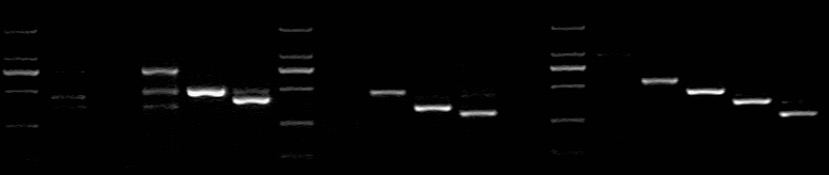

Supplement: S2 Fig — (JPG) [file pone.0305201.s002.jpg]

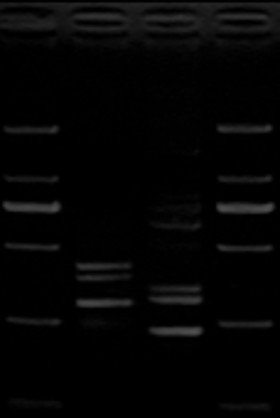

Supplement: S3 Fig — (JPG) [file pone.0305201.s003.jpg]

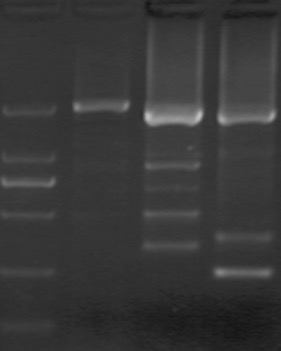

Supplement: S4 Fig — (JPG) [file pone.0305201.s004.jpg]

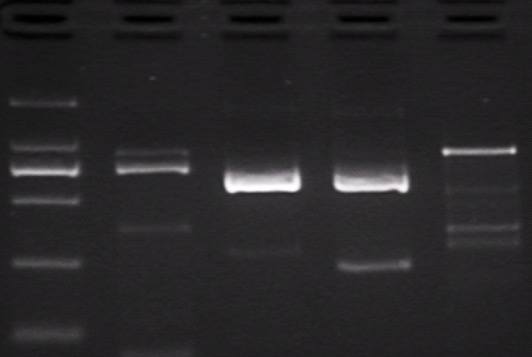

Supplement: S5 Fig — (JPG) [file pone.0305201.s005.jpg]
